# Binet’s Error: Developmental Change and Individual Differences in Intelligence Are Related to Different Mechanisms

**DOI:** 10.3390/jintelligence5020024

**Published:** 2017-06-09

**Authors:** Mike Anderson

**Affiliations:** School of Psychology & Exercise Science, Murdoch University, Murdoch 6150, Australia; Mike.Anderson@murdoch.edu.au; Tel.: +61-8-9360-2186

**Keywords:** IQ, mental age, cognitive mechanisms, speed of processing, executive functions, electroencephalogram (EEG) alpha, EEG theta

## Abstract

In common with most, if not all, papers in this special issue, I will argue that understanding the nature of developmental change and individual differences in intelligence requires a theory of the mechanisms underlying both factors. Insofar as these mechanisms constitute part of the fundamental architecture of cognition, this is also an exercise in unifying the discipline and research on intelligence in both children and adults. However, I argue that a variety of data support a theory suggesting that developmental change is the province of mechanisms commonly regarded as components of executive functioning or cognitive control, whereas individual differences are constrained by the speed of information processing. Perhaps paradoxically, this leads to the conclusion that Binet’s fundamental insight—that children’s increasing ability to solve problems of increasing difficulty could generate a single scale of intelligence—is wrong. Compounding the paradox, this means that mental age and IQ are not simply two different ways of expressing the same thing, but are related to two different dimensions of *g* itself.

## 1. A Brief History of the Conceptual and Measurement Basis of Intelligence in Children and Adults

The moniker “genius” should be used with great caution (especially in psychology), but few would doubt that Binet’s fundamental insight into the measurement of intelligence and the subsequent development of the intelligence test was a stroke of genius and arguably psychology’s greatest contribution to science in the 20th century [1]. What this stroke of genius was, is both fundamental to this journal’s special issue and not without irony. For the call of most, if not all, papers in this special issue is to unify the concept of intelligence as both an individual differences and a developmental construct—and yet this is exactly where Binet started over a century ago and this “unification” is the basis of his stroke of genius. But while, at the level of measurement, Binet did indeed lay the foundation for the development of intelligence tests and the unification of individual differences and developmental change, it has turned out that his basic insight was in error. This is because, I will argue, the mechanisms underlying individual differences and developmental change in intelligence are not only different but mutually exclusive.

## 2. Binet’s Genius

Intelligence tests have been with us for so long now that non-experts (that includes all psychologists not well versed in psychometrics) would often take for granted the sublime insight at the heart of the measurement of intelligence. Perhaps this is because the process of the construction of a scale of intelligence is well-known and relatively straightforward. Intelligence tests are composed of a series of questions, usually called “items” that test knowledge or knowledge acquisition. What makes intelligence tests special (and usually expensive!) is that this is not any “trivial pursuit” collection of questions but, rather, the items that are selected for an intelligence test exhibit special properties. Because Binet started from the premise that the nature of intelligence was to be found in the processes of reason and knowledge acquisition, the first place to start in measuring someone’s intelligence is to test their knowledge or ask them to reason their way through a problem. What becomes readily apparent, and this is the foundation of any test of intelligence, is that “items” that test knowledge or reasoning vary in their difficulty. Critically, the difficulty of an item is systematic, not whimsical, for otherwise the measurement of intelligence would be impossible. So it is relatively easy to determine an item’s difficulty. We simply note the proportion of times the item evokes a correct answer. This will be high for an “easy” item and low for a “hard” item. But this is just the first part of the process of item selection. 

The second stage is to ensure that the difficulty of an item reflects the difficulty of the scale overall. This again is accomplished relatively simply by determining if the probability of getting a particular item correct correlates with the probability of getting the other items correct, too. This means that only items showing positive correlations with each other will be selected as items for that measuring scale. Once we have selected the items for the scale, it now becomes possible to “invert” the empirical logic and define (measure) an individual’s ability (intelligence) by the number of correct items. The higher the score, the more intelligent the testee. What became immediately apparent to Binet was that the number of items that children get correct varies with their chronological age. Older children get more items correct—consistent with the pre-theoretical idea that older children are more “intelligent” than younger children. So far, all is straightforward but still theoretically empty. It is the next step that represents Binet’s stroke of genius. Because the ability to solve items is age-related in children, Binet had the idea of converting the scale into an age-normed scale where the norms relate to the scores typically achieved by children of particular ages. This allowed Binet to represent a child’s intelligence not by a particular score on a particular test but by a metric of “mental age” (MA)—mental age being equivalent to the chronological age (CA) for which that score would be the average. Thus Binet invented the construct of “mental age” and it has been the dominant construct in developmental considerations of intelligence. It was Wilhelm Stern [2] that doubled back on Binet’s procedure and expressed intelligence not as a mental age score, but a mental age score relative to a child’s chronological age—IQ = MA/CA × 100. Of course this classic formulation broke down in adults where mental age stops increasing somewhere between 14 and 18 (the Wechsler scales moved explicitly to calculating IQ as a deviation from the norm mean). Perhaps this should have been taken as a huge hint that the scales are actually different for children and adults.

And so it is that the score on a test can be indexed differently—typically either as a mental age in children or as IQ in adults. The consequence of this has been critical for the central theme of this journal’s special issue, for there is an implicit theoretical commitment in Binet’s formulation that most have taken for granted since, namely that mental age in children and IQ in adults, while different indices are fundamentally measures of the same thing—i.e., intelligence. But while the technology of developing tests has attained a reliability and sophistication probably unmatched in the rest of psychology, it is a weakness that the technology of test construction has advanced ahead of our theoretical understanding of the nature of intelligence itself. Indeed, understanding what makes one item more difficult than another (and what underlies the systematicity itself) and therefore the subsequent, but apposite, question of whether difficulty lies on the same dimension for children and adults, has progressed very little beyond the rudimentary. I will argue that when we come to consider the cognitive mechanisms underlying both the developmental and individual differences dimensions to intelligence we will find that, contra Binet, mental age and IQ are not simply different ways of expressing the same thing but are measures related to fundamentally different constructs based on variations in the functioning of different mental mechanisms.

## 3. The *g*-Factor

This is where the history takes another twist. Binet set the foundation of what a theory of intelligence should be about. At first pass, a theory of intelligence is a theory of cognitive “difficulty”; in short, what is it about our mental processing that makes one item more difficult than another. Binet and Simon, who are credited with creating the first intelligence test had a student, Jean Piaget, who famously led the charge on developing such a theory—at least in terms of cognitive development. But the rest of the field was distracted from developing a theory of cognitive difficulty by focusing instead on questions of structure—how many abilities are there and what best explains that pattern? Critical to this is the central empirical finding generated by the construction of intelligence tests—the discovery that cognitive abilities covary. That is, someone better than average on one cognitive ability measure will, in all likelihood, perform better than average on all the others, too. It was Spearman [3] who first named this phenomenon “*g*” or general intelligence. A key analytic technique, factor analysis, lay at the heart of this tradition and that story has been told so many times it need not be repeated here (for those wanting opposing views see [4] and [5]). Suffice to say that out of this came a near century-long dispute about whether Spearman’s construct of *g* was real, or the figment of statistical machinations. Even yet, the issue is not actually resolved [6] but rather a consensus has emerged, in psychometrics at least, in favor of the reality of *g*. Two major pieces of work, one a *tour de force* in factor analysis, and the other the harbinger of a new analytic methodology set to transform the scientific landscape.

The first was the hugely influential contribution of John Carroll, perhaps the foremost psychometrician of intelligence of his generation. Carroll [7], in a vast review of factor analytic studies of cognitive abilities, concluded that what people called Spearman’s *g* was the factor at the top of a hierarchical structure of cognitive abilities. Carroll’s approach laid the foundation for the major psychometric synthesis now broadly known as the Cattell–Horn–Carroll theory of fluid and crystallized *g* [8]. Fluid *g* is the ability to solve problems in the here and now, whereas crystallized *g* is the knowledge depository of the application of fluid *g* to the real world. Cattell [9] argued that these two kinds of *g* were differentially related to environmental and biological variables and have different developmental functions.

The second major influence on the debate was the development of latent trait analytic methodologies—now broadly known as confirmatory factor analysis. Gustafsson [10], using Lisrel to test the fit of different factor-analytic models on a database of 1000 individuals, showed that “fluid *g*” (a second-order factor in some models) was the same factor at the apex of the hierarchical structure of abilities where psychometric *g* is a single third-order factor (in other words, fluid *g* and psychometric *g* are one and the same “thing”). While it is gratifying that, at least for most of the relevant experts, this debate has now been put to bed in favor of *g* being a real phenomenon of human intelligence. I have argued for some time that to escape charges of solipsism (and perhaps worse, tautology) a theory of *g* cannot simply rest on the analysis of intelligence test data themselves, no matter how rigorous or technically clever [11]. This is because a theory of intelligence cannot itself be a theory born purely from a psychometric analysis of psychometric data, because a theory must speak to data beyond that which generated the theory in the first place [12]. Indeed, those so-called psychometric “theories” are not theories at all, but just more elaborate descriptions the data. Moreover, and more generally in psychology, we have known since the rise of information processing or cognitive psychology that a theory of intelligence must itself be constituted by a theory of the cognitive mechanisms underlying those observed differences we so exquisitely measure [11,13]. As we shall see, it is when we begin to specify the possible cognitive basis of *g* we see that it might be that Binet’s basic insight that individual differences in intelligence in adults maps onto mental-age differences in children, is wrong.

## 4. The Information Processing Basis of *g*

Historically, there have been two main contenders for the cognitive basis of general intelligence, or *g*. The oldest is that individual differences in speed of information processing would have an impact on all mental abilities having an information processing base. The second major contender in some ways arose out of criticisms of the evidential basis of the first, namely that individual differences in cognitive control (more usually referred to as executive functioning) provides the link across all task performance in ways that would result in a *g*-Factor.

The research program attributing *g* to differences in speed of processing (SoP) began in earnest in the 1970s with the reporting of correlations between relatively simple measures of reaction time and IQ [14]. A great deal of research showed that relatively simple measures of reaction time across many different kinds of information processing tasks were correlated with IQ. The argument was that the tasks were so “easy” that everyone could perform at 100% accuracy and that it was only how quickly they completed each task trial that was related to their intelligence. The analogy with a computer is direct. It was supposed that everyone performing these tasks utilized essentially the same cognitive algorithm (or computer program), but those with faster processors completed the task more quickly. Consequently, having a faster processor would create general differences in performance across all tasks that were based on computation (thinking) and lead to the phenomenon of general intelligence or *g*. It is important to realize that the evidence for the speed hypothesis depends critically on the belief that all task participants are running the same cognitive algorithm in the same way. However, this research program was criticized early, on the grounds that the information processing tasks themselves were not as cognitively “simple” as was at first claimed. Rather than mean reaction time differences on these tasks being the consequence of individual differences in the speed of processing of a single and simple cognitive algorithm, the counter argument was that the tasks presented a cognitive control problem—how to go fast without making too many errors. Thus, mean reaction times might better reflect individual differences in cognitive control and the selection of task strategies, rather than differences in speed of processing *per se* [15,16]. It was in this context that the correlation between IQ differences and an even “simpler” task, inspection time, became important. In a typical inspection time (IT) task participants have to make a simple perceptual discrimination, usually whether two lines are of unequal or unequal lengths or which of two lines is the longest. The exposure duration of the stimulus is varied by using a backward mask that prevents further processing of the IT stimulus. The exposure duration of the stimulus is varied systematically allowing an estimate of an accuracy threshold (usually about 70%). This threshold is an individual’s inspection time and is taken to index their speed of information processing. Shorter inspection times, therefore reflect, higher speed of information processing. A number of reviews and meta-analyses have confirmed that the likely correlation between inspection time and IQ may be around 0.5 in the general population [17,18,19]. This is a very high correlation for a single task measure and is one of the main lines of argument that supports the claim that variations in speed of processing causes individual differences in intelligence. Yet even the inspection time task has been subject to criticisms that the task is subject to strategic variation and that the selection of optimal processing strategies may be the basis of its correlation with IQ [20]. It is also true to say that the speed of processing hypothesis failed to gain momentum because it was still not clear what the processing architecture was in which speed was embedded. This is another way of saying that most of the “speed” hypotheses of *g* are but statements of association and appeals to common sense (faster must be better) since there is no detail of how speed effects some processes and not others (which requires a commitment to a specific cognitive architecture). By contrast the cognitive control literature started from an explicit architecture, namely that proposed for the structure of executive functioning.

## 5. Executive Functioning and Intelligence

Executive functions (EF) can be broadly defined as those processes that afford the flow of control in information processing. Although there are other theories of the structure of executive functions, the structure proposed by Miyake et al. [21] is widely cited. Miyake proposed that there are three EFs: (1) inhibition, or the suppression of prepotent or interfering responses or stimuli; (2) shifting, or the ability to switch between tasks or mental sets; and (3) updating, others usually simply refer to this as working memory. These functions are all lower-order executive functions considered to be involved in a range of other higher-order functions, and that multiple measures can tap each function. Miyake et al. [21] used confirmatory factor analysis (CFA) to create latent variables for each of the three executive functions and found that they were all related (unity), yet separable (diversity). The unity between the constructs was evidenced by moderately strong correlations between each latent variable (range *r* = 0.42 to *r* = 0.63), but having three separate latent variables provided the best model fit for the data, which justified the claim for discriminant validity (diversity) of the three EFs. Friedman et al. [22] extended the Miyake et al. [21] model of executive functions to determine which predicted fluid and crystallized intelligence (gF and gC respectively), and found that only updating (or working memory) predicted the two intelligence constructs. However, many other studies have found a strong enough relationship between intelligence and measures of inhibition, working memory, set-shifting and the like to lend substance to the claim that they all might contribute to the information processing basis of *g*.

As such, we have two possible candidate processes for the cognitive basis of *g*—speed of processing and executive functioning—and a reasonable claim, following Binet, is that one or both of them (that is, both in equal measure for children and adults) contribute to individual differences and to developmental differences in intelligence. Indeed, it is well established that EF and SoP are associated with intelligence in children [11,13,23] but what can we learn from the specific pattern of association with each other and with fluid intelligence and mental age?

## 6. Development of Speed of Processing (SoP)

Given the strong association between speed of processing and IQ in adult research, it was a reasonable step for those interested in the development of intelligence to propose that perhaps this is underlain by changing speed of processing (SoP). At first blush, there is a great deal of evidence in favor of this idea: (1) The two major methods of measuring SoP, reaction time and inspection time, both show substantial improvements with [24,25,26]. Developmentally, reaction time measures of task performance show a regular decrease across a variety of information processing domains, for example letter-name retrieval [25,27], mental rotation [27,28]), memory search [25,27], and numerical calculation [28]. Inspection time also has been shown to decrease with age during development [26], although not as strongly as reaction time [29]. (2) Brinley plots, where mean reaction times for many different kinds of information processing conditions are compared across different age groups, seem to reveal amazingly linear functions suggesting a common (or general) developmental factor that many have interpreted as changing SoP for both child development [27,28,30,31,32,33,34] and cognitive decline in ageing [35,36,37]. However, I have long disputed this interpretation for both reaction time and inspection time data [11,38,39], and for Brinley plots [39]. In the case of reaction time and inspection time, I argued that both of these tasks might have processes that are unrelated to IQ in adults but are sensitive to developmental changes in children. It is also noteworthy that even apparently “simple” tasks like inspection time show changes as a function of experience, although the effect of experience and maturation can be separated with appropriate research designs [40]. The fact that there are experiential effects on tasks like inspection time is further demonstration of the pitfalls in taking an operational stance (i.e., that inspection time *is* SoP and therefore changing inspection time with age is evidence for changing SoP). But what components of tasks like inspection time and reaction time might be subject to maturational, rather than experiential, change with age? As an example, Anderson et al. [41] compared reaction times in seven- and 11-year-old children under two task conditions with different response selection demands. When speed estimates were derived from these tasks, they, found that only the condition with high response selection demands show apparent changes in speed with age. Even in inspection time tasks, there are response selection components that impose a relatively greater load on younger children [42]. I have argued, then, that what changes with age in these tasks for children is not SoP *per se* but response selection processes that contribute more to task performance in children than they do in adults. While it may be appealing to parsimony to argue that speed underlies both individual differences in intelligences and developmental changes, some behavioral data are consistent with the alternative view. For example, in the case of SoP, Anderson [43] showed that when RT tasks (in theory, for children, more “loaded” on executive processes, though they have speed components, too) are contrasted with IT tasks (more loaded on speed and less loaded on EF processes) we find that even in children, IT is more related to IQ but RT is more related to mental age. The interesting interpretation that reconciles both the data on SoP and that of executive functioning (as we shall see), is that the former are related to individual differences and the latter to changes in mental age. Indeed an older literature on mental age matching [44], demonstrates that counter to Binet’s view, equating mental age does not render cognitive equivalence with children of higher IQs, but the same mental age as children of lower IQ having different cognitive strengths and weakness. This belies the claim that mental age and IQ represent the same dimension of general intelligence.

## 7. The Development of Executive Functioning

There is no doubt that the measures of the many facets of executive functioning, principally: working memory; inhibition; set-shifting; error monitoring; goal setting and goal maintenance, undergo considerable development change. This makes executive functioning the prime candidate for the process underlying the development of general intelligence. In particular, the notion of working memory capacity, so popular in developmental circles today, was pre-dated by the older more generic construct of processing capacity—was offered as the driver of general developmental change by theorists such as Pascual-Leone [45] and Halford [46]. Both theories argue that the development of intelligence involves the child’s increasing ability to cope with the demands on cognitive capacity imposed by problems of increasing structural complexity. Halford’s theory supposes that increases in cognitive capacity enable the older child to hold task representations of much higher complexity in mind than that within the capabilities of a younger child. This increasing capacity, couched now in working memory terms, would allow the developing child to achieve much better executive control in many problem-solving situations. In this tradition, Demetriou and colleagues [23,47] have attempted to tease apart the influence of SoP and working memory as well as other executive functions at different points of development. They have a complex developmental model that has proposed shifts and cycles in development where processes of representational change interact with the development of some basic information processing capacities (such as speed, working memory and inhibition). Indeed some basic capacities like speed are seen as consequences rather than causes of representational and developmental changes. Equally this makes it problematic to obtain clear (never mind pure) measures of these constructs, if one level (representational) can influence apparent capacity at another level (e.g., speed). It then becomes difficult to disentangle one construct and one measure from another.

While it may be the case that most developmentalists hold that some kind of changing cognitive capacity underlies development, some others have argued that it is a particular “frontal function”, namely inhibition, that causes this change in capacity. Again it is not difficult to find research that demonstrates that older children appear to be better at inhibition in a number of the classic paradigms (Stroop, go-no-go, stop RT, etc.). Bjorklund & Harnishfeger [48] in particular put great store in the development of inhibition as the general motor of developmental change. We have also argued that inhibitory processes may be core to developmental changes in fluid intelligence although this argument has recently met with mixed empirical support at best [49]. Inevitably other theorists want to claim that one of the other EF processes (for example, working memory) is the critical dimension of EF that is related to intelligence. And so it goes. Whatever the underlying cause, all agree that children can solve problems of increasing difficulty and that their apparent cognitive capacity increases during development. So even if it is only at the level of description, it is uncontentious to regard the development of general intelligence as being correlated with the development of executive functions.

The consideration of SoP on the one hand and executive functions on the other as the mechanistic basis of differences in *g*—whether as an individual differences or a developmental construct—has been considered largely independently of one another or as equivalent contributors, at best, to intelligence at any stage on the developmental spectrum [23,47]. However, Anderson’s theory of the minimal cognitive architecture underlying intelligence and cognitive development [11,13,50], posits that SoP and EF are two separate dimensions of general intelligence but, more than this, speed is related to individual differences (IQ) and EFs are related to developmental change (mental age).

## 8. The Theory of the Minimal Cognitive Architecture (MCA) of Intelligence and Development

**Figure 1:**
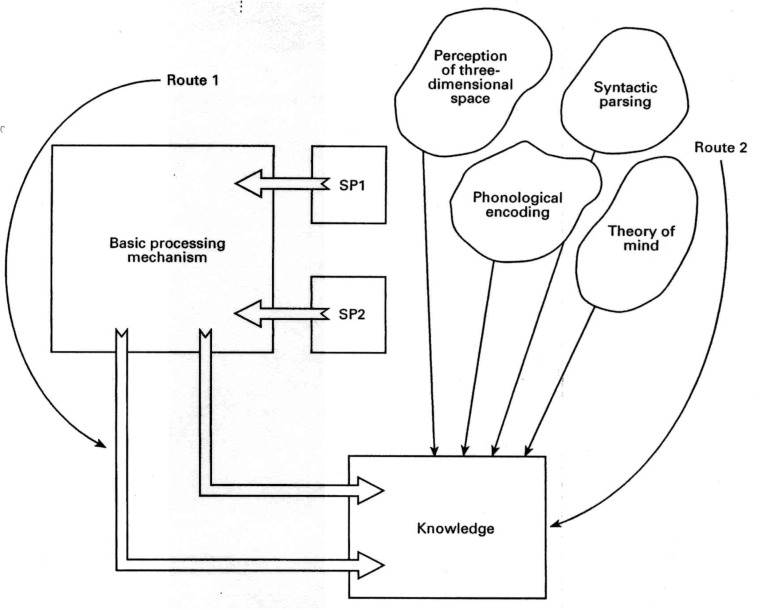
The theory of the minimal cognitive architecture underlying intelligence and development. Reproduced with permission from Anderson, M. [11] (p. 107), published by Wiley-Blackwell, 1992.

Briefly, minimal cognitive architecture (MCA) theory states that there are two routes to knowledge (see Figure 1). The first route (thinking) uses general problem-solving algorithms generated within two specific processors, SP1 and SP2, which are dedicated to verbal and visuospatial processing, respectively. The algorithms that can be generated by each specific processor define its latent ability. However, it is speed of processing (SoP), determined by a basic processing mechanism (BPM) that constrains the complexity of algorithms that may be implemented (the SP’s manifest ability). Thus speed causes the manifest abilities of the two processors to become correlated and so it is this constraint that is the fount of *g*. This first route is equivalent to gaining knowledge through thought. As such, the speed of the basic processing generates *g* and increasing speed increases intelligence. A novel hypothesis of this theory is that SoP does not change with development. This means that developmental change and individual differences are *necessarily* two independent dimensions of *g* in this theory. That SoP is unchanging with development also brings with it an explanation for the relative stability of IQ differences across years of considerable change in functioning intelligence (or mental age). It is the second route to knowledge acquisition the theory claims is subject to major developmental changes (see Figure 1).

The second route to knowledge involves the use of cognitive modules [51]. Anderson described several types of modules that include Fodorian-like modules that are genetically pre-specified and present from birth, as well as those that emerge as a consequence of experience. While the latter are likely to be created by extended experience (or practice) in particular domains, the former underpin developmental capacities acquired early in life and are unlikely to have much influence on intellectual development in school-aged children—other than in cases where a module might be damaged [52,53,54]. On the other hand, a third set of modules are the “fetch-and-carry” mechanisms of information processing (or as Pylyshyn [55] put it, part of the functional architecture of cognition) that probably follow protracted developmental trajectories and consequently influence the development of intelligence itself. The cognitive mechanisms that are the components of executive functioning and cognitive control fall under this category. Thus, within this theory, executive functions are determined by mechanisms that are modular in the architecture and consequently unconstrained (and therefore uncorrelated) with the speed of information processing. Accordingly, modular information processing represents a second and uniquely developmental dimension to *g*.

In short, Anderson [11,13,38] has proposed that in development, IQ and mental age are the products of two distinct (albeit interrelated) processing systems. Individual differences are related to SoP, which is unchanging in development, whereas developmental changes are largely driven by the development of modular systems, particularly when they support the cognitive control/executive functioning processes. In other words, this theory argues that Binet was indeed in error because the mechanistic basis of individual differences in *g* and developmental change in *g* are not only different but mutually exclusive. I should be clear in this context what is meant by mutually exclusive. At one level, this simply draws attention to the fact that the mechanistic basis of individual differences in SoP is different to that of the mechanistic basis of developmental changes in EF and that it is the latter that drives cognitive change (in the theory this *must* be, because SoP is unchanging). Of course in the developing mind there will be a synergistic interaction between a child’s SoP and its EF maturity that will determine the difficulty of cognitive problems that the child can accommodate.

In this architecture, SoP and EF are subsumed by different processing mechanisms and therefore constitute independent dimensions of *g*; however, in any cognitive task these processes work in concert and consequently are difficult to disentangle. One way to do this is to look at the relative loadings of various speed and cognitive control components in different tasks and predict those that are more related to “true” SoP (these will correlate more strongly with IQ across development), and those more related to EF will be more strongly related to mental age. This prediction was supported in the study by Anderson [29] described above and the influence of different processes (for example, speed, attention and response section) has been explored in a number of experimental contexts [41,43,50,56]. Another approach has been to look over development at the structure of EF and how it might relate to intelligence. Brydges et al. [57] conducted confirmatory factor analysis (CFA) and structural equation modelling (SEM) on a sample of 215 seven- and nine-year-old children, and found that a unitary EF factor (as opposed to a diverse collection of executive functions) was highly predictive of both fluid and crystallized intelligence. In another longitudinal study, they showed that the factor structure of EFs themselves changed between the ages of seven and 11 [58], and finally that there were precursors of component processes in EF to be found in ERP (N2) data before differentiation of functions at the behavioral level could be found [59]. Clearly, if EFs develop and unfold over time, their contribution to intelligence must differ at different ages and to this extent, at the very least, represent a different dimension to intelligence from that contributed by SoP (see [60] for an alternative view).

While a number of experimental studies have attempted to test the specific hypothesis that speed is more related to IQ, and cognitive control, or executive functioning (EF), to mental age in children the theory can also generate a number of psychometric predictions related to other questions of interest in the development of intelligence. An example offering the possibility of another potential test of whether speed changes with development is to model theoretical predictions concerning what is known as the “differentiation hypothesis”.

## 9. The Differentiation of Abilities

Anderson and Nelson [61] generated a series of psychometric predictions developed from the theory of the minimal cognitive architecture that are related to the differentiations hypothesis. As they pointed out, the differentiation hypothesis actually comes in two varieties that fit rather nicely with the theme of this special issue: (1) individual difference differentiation—this hypothesizes that as measured ability goes from high to low in same-age individuals, specific abilities will become more differentiated and *g* will account for less of the total cognitive ability variation; (2) developmental differentiation where specific abilities become more differentiated during child development and maybe less so through ageing (de-differentiation). Because in the theory of the MCA SoP constrains the manifest abilities of the two specific processors (SP1 and SP2 above, see Figure 1), this has a “natural” consequence that differences in the latent abilities of these processors will become more manifest at higher speed of processing (SoP). If SoP is in turn the fount of *g* this means that specific abilities will become more differentiated at higher *g* (IQ). So ability differentiation comes “free” with this theory. Modelling the theory, Anderson and Nelson tested what might happen given a number of alternative developmental options. The option they favored was where the specific processors (SP1 and SP2) develop but the SoP is unchanging. A simple model was used to generate hypothetical scores generated on 10 tests symmetrically but unequally loaded on the manifest ability of each specific processor. 

The basic form of the model rested on the constraint imposed by SoP and the “latent” ability of a specific processor (SP_l_) on the manifest (measurable) ability (SP_m_) of that specific processor, thus:

SP_m_ = log(SP_l_)

With two specific processors, each with a manifest ability, a hypothetical test score can be generated for each individual on a number of tests. These tests were designed to load equally and symmetrically on each specific processor. Forty-eight percent of the variance of the first test was determined by SP1 and 2% by SP2 through SP2 (with 50% of the residual variance attributable to measurement error), and the middle two tests had 30% and 20% attributable to SP1 and SP2 (and vice versa) until finally test 10 was the converse of test 1 with 2% of the variance attributable to SP1 and 48% attributable to SP2. Another interesting feature of this modeling is that SoP is not explicitly represented in test scores—only implicitly on the constraint it imposes on the specific processors. These scores were generated for 5000 hypothetical individuals whose SoP, SP1_l_, and SP2_l_ were all normally distributed and uncorrelated with each other. 

Differentiation was then tested by measuring: (a) the size of the first principal component of the resultant test battery (a proxy for *g*); (b) the average size of the intercorrelations amongst the tests; and (c) the size of, the second principal component from the test battery. The model simulations pointed strongly to individual differences differentiation and somewhat surprisingly, this was most marked not by the size of the first principle component but by the size of the second. The models also pointed to a relative lack of differentiation over child development. Subsequently these models were compared to a real dataset of 181 children aged between eight and 12 years old for whom we had scores on the Cattell Culture Fair (scale 2, form A) plus nine subtests from the Wechsler Intelligence Scale for Children (WISC) III. Consistent with the favored developmental model there was very little evidence for increasing differentiation with age in this sample but there was marked differentiation when the sample was split on IQ and surprisingly there was an indication that there might be more ability differentiation for younger than for older children. Although not consistent with the favored view that child development represents a period of increasing differentiation of abilities, it is consistent with the idea that, if anything, *g* becomes more dominant in later years than in early years.

## 10. Where to from here?

Despite the kinds of evidence above, many remain unconvinced that SoP and EF represent independent dimensions to intelligence and more particularly that one process (e.g., SoP) or other (e.g., working memory) might underpin a different dimension of fluid *g* (see for example [62,63]). In part this is unsurprising because there is lack of behavioral-task purity—i.e., there is no candidate task that measures only one of those processes and one alone. It is very difficult to extract the processing components of compound tasks and unambiguously assign them to either construct. Consequently, alternative theories coexist with no uncontentious knockout empirical data to discriminate them. However, one obvious development of the last 10 years has been the growing influence of neuroscientific accounts of cognitive control and how this may be related to fluid intelligence. The question is, might the deciding evidence for alternative theories of intelligence and its development be found in neurophysiology or neuroanatomy? Despite concerns about naïve reductionism [64], I think it just might be so.

## 11. Duncan’s Multiple Demand Theory of Fluid *g*

Duncan [65,66,67] has argued that Spearman’s *g* and frontal processes are synonymous. The motive for his theory was provided by the paradox that while the frontal cortex of the brain has often been considered the seat of human intelligence, damage to the frontal regions often leave a patient’s IQ unaffected. Duncan’s resolution of this paradox was to point out that intelligence tests measure two important aspects of general intelligence, fluid and crystallized *g*. Duncan argues that the frontal lobes of the brain are the areas responsible for instantiating cognitive routines for problem solving. These routines involve the establishing of hierarchies of task goals, maintaining those goals and monitoring ongoing information processing in service of those goals—the core functions of what others call executive functioning. Duncan developed a task to measure goal neglect—a phenomenon seen in patients with frontal damage. This task requires participants to perform in accordance with a series of relatively simple instructions. For example, they are instructed to monitor a rapid serial visual display of pairs of letters and digits and can be asked to: (1) report the letter only from the right-hand side of the pair; (2) ignore numbers and (3) “switch” side on the appearance of one of two possible cues. Stringent tests ensure that the participants both understand and remember these instructions, but nevertheless they often fail to comply (goal neglect). In the goal-neglect task, Duncan not only showed that patients with frontal damage performed very poorly, but that performance on this task in individuals with no known brain damage is predicted by their levels of fluid *g*. Thus Duncan’s claim is that frontal damage affects fluid, but not crystallized, *g* leaving the largely crystallized measures of their IQ unaffected. Using a variety of experimental tasks and a variety of neurophysiological data, Duncan [68] has demonstrated that fluid *g* is highly correlated with activity and integrity of a frontoparietal network. Duncan’s Multiple Demand (MD) theory is really the first of a new approach to understanding *g*. It is cognitively sophisticated; that is, it does not just rely on psychometric g as a variable in a correlation, rather there is a theory of the cognitive mechanisms underlying the tasks used to measure *g* behaviorally and it is well-grounded in a detailed theory of how this cognitive system is implemented in the brain [69]. So instead of simply observing correlations of gross brain dimensions (e.g., gray-matter thickness) with any psychometric measure of *g*, it makes precise predictions about under what kinds of task conditions, and using what kind of indices of the functioning or integrity of the fronto-parietal network we might to expect, (or not expect), to find correlations with *g*. Is it possible that by adding such a neurodevelopmental dimension to our cognitive theories of *g* we might find some new data that might support or refute one theory or another? I tentatively suggest two candidates for the measurement of the brain-basis of both SoP and cognitive control or EF that would lead to predictions that might not be testable at the behavioral level alone.

## 12. A Neurodevelopmental Speculation

There is a large body of literature linking EEG alpha and theta frequency bands directly to *g* [70,71,72,73]. The alpha band refers to oscillations in the EEG at 8–12 Hz and theta at 4–8 Hz. Such frequencies are taken to reflect the concerted activity of neurons engaged in particular cognitive or perceptual activities. There is some evidence to suggest that individual differences in SoP might be related to differences in the alpha frequency band generated by cognitive activity. Bursts of alpha can be generated during tasks that require stimulus processing and alpha-phase has been shown to be related to optimal stimulus processing [74]), related to SoP [75]), and resting alpha has been shown to be correlated with intelligence [76]. A key research development would be to link both inspection time performance and intelligence to alpha phase (or resting state).

While EEG alpha is looking promising as a marker of neuronal activity associated with SoP so there is also a evidence linking EEG midline frontal theta to working memory and cognitive control, or executive functioning [77], and with intelligence [78]. A recent study by Roberts & Anderson [79] provides an example of how the combination of a detailed theory of cognitive functioning and its relation to intelligence, coupled with experimental manipulations and predictions about EEG signatures might provide powerful new ways to test and differentiate theories about the underlying basis of *g*. This study used a task (goal neglect) and an experimental manipulation (varying instruction load) first suggested by Duncan et al. [80] (but modified for children) and predicted that goal neglect would be influenced by instruction load and that this would be reflected in increased midline frontal theta in a specific condition. These predictions were borne out. In addition, the prediction was made that a higher correlation with *g* would be found in the task condition that evoked more goal neglect and increased midline frontal theta. This too was borne out.

Even more speculatively, it might be that SoP/alpha and frontal midline theta are, themselves, related to structural properties of the brain. One hypothesis is that SoP may be indexed by EEG alpha and that, in turn, may be related to dimensions of white matter integrity. There is some tentative encouragement for this hypothesis. Koenis et al. [81] found evidence for relationships between both global and local matter integrity and intelligence across the cortex. Recent studies of healthy older adults have found an association between white matter integrity and SoP [82]. Similarly we might hypothesize that developmental *g* is related to cognitive control, may be indexed by EEG midline frontal theta and is the consequence of grey-matter density in the frontoparietal network that Duncan claims underpins differences in *g*. And the central argument of this paper—that SoP and EF represent two different dimensions of *g*—is at least consistent with the observation that white and grey matter development follow quite different trajectories—with the former increasing linearly through childhood and adolescence, and the latter following inverted U-shaped curves [83]. And while the association of white matter integrity with intelligence seems to be found across the brain, the association between connectivity measures and *g* seem higher for specific regions of the brain such as lateral prefrontal cortex, that are specific to fluid rather than crystallized versions of *g* [84].

A very promising methodological and theoretical approach to what is ultimately a complex causal chain from underlying neuronal processing through interlevels of cognitive systems to psychometric and behavioral levels of analysis, is the combination of a taxonomy of endophenotypes with theoretically driven hypotheses of causal links between them and “watershed” models that acknowledge that the resultant behavior of interest (in our case individual differences and the development of intelligence) can have multiple causes with multiple pathways [85]. A good example is provided by Kievit et al. [86] who applied a structural equation modelling (SEM) analysis to test alternative models of the cause of individual differences in fluid intelligence in a sample of adults from 18 to 87 years of age. Their analysis incorporated a number of measures of white matter integrity using a number of measures derived from fractional anisotropy, a number of cognitive measures of speed of information processing and psychometric measures of fluid intelligence. They found the best fitting model was one of a “many-to-one” relationship between SoP and fluid intelligence in this sample, suggesting that SoP may not represent a single pathway to fluid intelligence and, moreover, that there may be different kinds of speed. While they did find strong relationships between their white matter measures and SoP, they were not uniform across the speed measures. And while they found support for a hierarchical relationship between white matter integrity, SoP and fluid intelligence (as predicted by their watershed model) there was no evidence of a single pathway between them. The authors speculate that cognitive measures of working memory may increase the fit of their model and acknowledge that much may depend on the individual measures used to derive the latent traits in the model.

**Figure 2:**
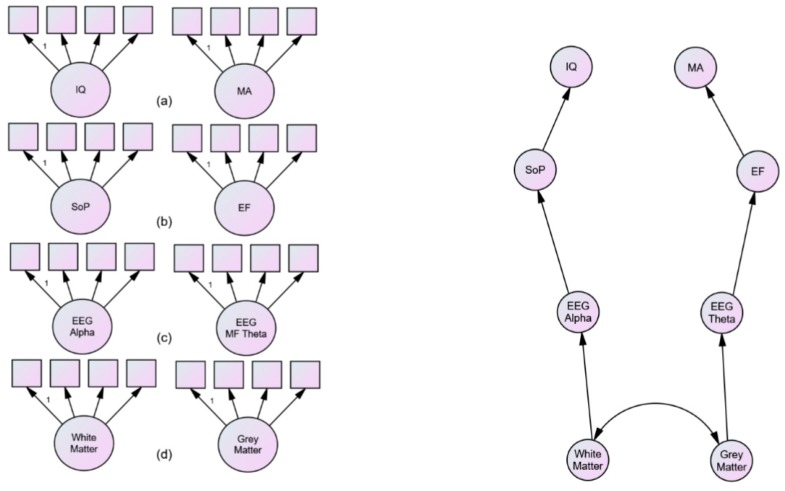
A SEM approach to testing causal models of the endophenotypes of intelligence. The first panel shows four measurement models at different levels of endophenotypic description: (**a**) latent trait of (age-normed) IQ differences from intelligence tests contrasted with latent trait of (age-related) indices of mental age; (**b**) latent trait of indices of EEG alpha contrasted with latent trait of indices of frontal midline theta activity; (**c**) latent trait of cognitive/information processing measures of speed of processing contrasted with latent trait of indices of cognitive measures of executive functioning; (**d**) latent trait of indices of white matter integrity contrasted with latent trait measures of frontal grey matter morphology. The second panel is a schematic of endophenotypic pathways with one from white matter integrity, through EEG alpha, through SoP to IQ differences and the other from frontal grey matter, through EEG frontal midline theta, through EF to mental age.

While the results of the Kievit et al. [86] study are not conducive to the theoretical stance taken in this paper, the approach is worth noting. Isolating single factor congeneric latent traits for each of our constructs at each of our endophenotypic levels will be crucial for testing our models (see Figure 2, panel 1). Moreover, our research design will have to be sensitive to both the sample (age, IQ, mental age). For those of us interested in developmental change *per se*, it should incorporate both cross-sectional and longitudinal samples to allow us to tease apart the role of experience from potential maturational changes at each level of our endophenotypes and subsequently to test causal models of the effect of each one on the others (see Anderson, Reid and Nelson [40] and Bishop et al. [87]) for examples of such a design). The essence of this approach and how it might apply to the cognitive theory presented in this paper and its neurodevelopmental speculation is illustrated schematically in Figure 2. Panel one illustrates that we should adopt an SEM approach where we develop single traits for our constructs from measures that themselves have a justifiable status as indices that of the traits we are interested in (in my book, many papers using psychometric, or paper and pencil, indices of SoP are no such thing). And we should do so in a context where we are trying to contrast at least two possible dimensions or pathways in our causal models—as such, finding variables maximizing the distinction between SoP and EF. Panel 2 presents a hypothetical hierarchical pathway for our endophenotypes and represents a model derived from the theory proposed here—where a single pathway though the cognitive endophenotype—SoP, causes individual differences in IQ, and a separate pathway through the cognitive endophenotype—EF, causes differences in mental age. It should be noted that this represents a very strong model because there are many other possible pathways though the hierarchy and therefore this should be a theory that can be easily falsified if it is not true. Ultimately using an appropriate cross-sectional/longitudinal design enabling us to distinguish experiential from maturational change can allow us to measure any maturational change in mental age. The prediction from the theory advocated in this paper would be that this maturational change would be predicted by EF and not by SoP. 

## 13. Conclusions

The main contenders for the mechanistic basis of *g* are SoP and the various components of EF. There are many studies lending support to each but very few that attempt to pit one against the other or ask the question of whether their relationship is different for children and adults. As a field, we are still coming to terms with Binet’s basic insight that changing cognitive ability in children can be used to construct a scale of intelligence (or cognitive difficulty) that can also measure intelligence in adults. In the century since Binet’s seminal work, research on intelligence in adults and children has gone in quite different directions. But this paper argues that there is a deep reason for this: Binet was in error. Individual differences in intelligence in adults and developmental change in children are underpinned by different mechanisms (SoP in the former and EF for the latter). This is far from accepted in the field and part of the problem may be that behavioral tasks and the indices developed from them are not pure in process. Perhaps theories linking psychometric *g* with underlying cognitive mechanisms *and* with neural sequalae using neuroimaging techniques and the analytic methods of SEM within a watershed model approach might prove to be definitive on this question. And of course, Binet did not have the advantage of either a cognitive framework or modern neurodevelopmental measurement on which to base his ideas. If he is proved wrong in the long run, he is surely and respectfully forgiven.
